# Prevalence and incidence of attention-deficit/hyperactivity disorder in Slovenian children and adolescents: a database study from a national perspective

**DOI:** 10.3325/cmj.2015.56.159

**Published:** 2015-04

**Authors:** Matej Štuhec, Vesna Švab, Igor Locatelli

**Affiliations:** 1 Clinical Pharmacy Department, Ormož Psychiatric Hospital, Slovenia, Ormož, Slovenia; 2 Department of Psychiatry, Faculty of Medicine, University Ljubljana, Ljubljana, Slovenia; 3 Chair of Social Pharmacy, Faculty of Pharmacy, University of Ljubljana, Ljubljana, Slovenia

## Abstract

**Aim:**

To estimate prevalence and incidence of attention deficit hyperactivity disorder (ADHD) in children and adolescents in Slovenia using different epidemiological models.

**Methods:**

Data from the National Institute of Public Health of the Republic of Slovenia for the period 1997-2012 were analyzed. The database includes the annual number of newly diagnosed outpatients with ADHD in Slovenia. The evaluation for ADHD diagnoses was done in accordance with the Tenth Revision of the International Classification of Diseases (ICD-10) outpatient data codes. In model 1, a linear increase was proposed to fit the data in the period from 1997 to 2003 in order to extrapolate the data before 1997. In model 2 and 3, an exponential increase in the annual incidence rate was proposed.

**Results:**

The incidence rate of ADHD diagnosis in 1997 was 0.032% and in 2012 it increased to 0.082%. Mean prevalence rate was 750 (95% confidence interval: 660-840) per 100 000 children and adolescents. It was estimated that the prevalence rate in 2020 would be 1% (95% confidence interval: 0.875-1.125), which is 6.3-fold higher than in 1997.

**Conclusions:**

ADHD is a common mental health disorder among Slovenian children and adolescents, but it remained underdiagnosed compared with Western countries. Our results indicated a need for improved timely interventions in Slovenia, not only in child and adolescent psychiatry but also in primary settings and adult psychiatry, where ADHD should be more efficiently recognized.

Attention deficit hyperactivity disorder (ADHD) is one of the most common psychiatric disorders in childhood and adolescence, characterized by developmentally inappropriate inattention, hyperactivity, and/or impulsivity (1). It entails economic costs, causes family stress and academic and vocational adversity, and has a negative effect on the patient’s self-esteem (2). Although it is seen as a problem that takes place predominantly in childhood and adolescence, clinical and epidemiological research has shown that in 30–50% of patients ADHD persists into adulthood (3,4).

Children exhibiting symptoms of inattention, hyperactivity, and impulsivity have been described previously (5). In 1980, the third edition of Diagnostic and Statistical Manual of Mental Disorders (DSM-III) introduced the term “ADD (Attention-Deficit Disorder) with or without hyperactivity.” DSM-III-R (1987) replaced this term by the term ADHD and DSM-IV presented the subtypes of ADHD (6-9).

ADHD affects 3% to 9% of children worldwide (10). In European countries, it is identified as a hyperkinetic disorder according to the 10th Revision of the International Statistical Classification of Diseases and Related Health Problems (ICD-10) and in North America as ADHD (DSM-IV). DSM-IV criteria include a broader group of subjects than the ICD-10 criteria (11,12).

While the popular press across Europe frequently comments on increased rates of ADHD diagnosis, questioning whether ADHD is overdiagnosed and overtreated, reviews of clinical practice suggest that in Europe ADHD is underdiagnosed and undertreated (13,14). ADHD is underdiagnosed especially in adults; which is why it is important to better understand the factors that contribute to accurate diagnosis (15). Diagnosis and management of ADHD include nonpharmacological treatment, including behavioral therapy, and pharmacological treatment with stimulants and nonstimulants (16). In many European countries including Slovenia, the incidence and prevalence of this disorder are not well researched. In Slovenia, there are also no national guidelines for ADHD treatment and diagnosis and no published data on the prevalence of ADHD (17,18). The primary aim of this study was to calculate the incidence and prevalence of ADHD diagnosis among children and adolescents in Slovenia in 2012, based on national data from 1997 to 2012. For this purpose, several different epidemiological models were developed. The secondary aim was to predict the number of Slovenian children and adolescents that will be diagnosed with ADHD in 2020.

## Methods

### Data collection

The national epidemiological data were obtained from database of the National Institute of Public Health of the Republic of Slovenia (NIPH) (19). The database includes information on the annual number of new patients diagnosed with ADHD in Slovenia from 1997 onwards, and their sociodemographic data. In Slovenia, the diagnosis of ADHD in children and adolescents can be made only by psychiatrists with specialization in child and adolescent psychiatry. At the first visit to the family physician after the diagnosis, the patient gets a diagnosis record, which is included in the database. The required population data were obtained from the Statistical Office of the Republic of Slovenia (20).

This study included the data regarding the following diagnoses: hyperkinetic disorders (F90), disturbance of activity and attention (F90.0), hyperkinetic conduct disorder (F90.1), other hyperkinetic disorders (F90.8), and unspecified hyperkinetic disorder (F90.9). The participants’ selection was limited to those younger than 19 years. Ethical approval was received from the National Medical Ethics Committee of the Republic of Slovenia.

### Modeling the annual incidence rate of ADHD diagnosis

The estimate of annual incidence rate was calculated as the ratio of the number of newly diagnosed children and adolescents with ADHD and the total number of people younger than 19 years. As the data regarding the number of newly diagnosed patients with ADHD had been collected only from 1997 onward, the incidence rate of ADHD diagnosis was modeled in order to estimate the incidence rate before 1997. The annual incidence rate was increasing in the period from 1997 to 2003, reaching a plateau from 2004 to 2012.

In model 1, in order to extrapolate the data before 1997, it was assumed that in the period from 1997 to 2003 there was a linear increase in incidence (for the purpose of the study, we assumed that the incidence rate before 1997 was zero). A linear regression model (Eq.1) was used to calculate the slope of the line (*b*), presenting the increase in annual incidence rate (*I*) per one year. The intercept (*a*) represents the estimated annual incidence rate in 1997 as the variable *t* (years) in Eq 1 was set to zero for the year 1997:


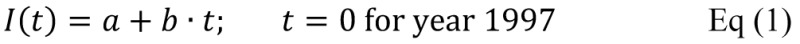
Eq [1]

In the model 2 and 3, an exponential increase was assumed. An exponential regression model was used to estimate the rate constant (*k*) of incidence rate increase and the intercept *A*, which represents the estimated annual incidence rate for 1997:


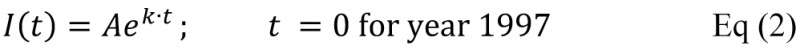
Eq [2]

The exponential model approximates to zero incidence rate, thus two starting points were selected. For model 2, the starting point was set to 1987 as in this year ADD was replaced by ADHD in the DSM-III-R (8). For model 3, the starting point was set to 1980 as in this year ADD, with or without hyperactivity, was introduced to the DSM-III (7).

We also estimated the prevalence rate without the extrapolation of the annual incidence rate before 1997 (raw data approach). In this approach it was assumed that in Slovenia no ADHD diagnosis existed before 1997.

### The estimation of prevalence rate of ADHD diagnosis

Estimate of prevalence rate (*P*) of ADHD diagnosis among children and adolescents was calculated on the basis of the estimate of annual incidence rate (*I*) and the proportion of patients who reached the age of 19 each year (*G*), using a recursive formula:


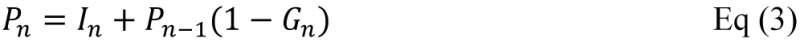
Eq [3]

where index *n* denotes the specific year (eg, 2012). It was assumed that the percentage of patients with ADHD who annually reached the age of 19 equaled to the ratio of the number of all 19-year-olds and the total number of all people aged 19 or younger. The required population data for the period from 1997 to 2013 were obtained from the Statistical Office of the Republic of Slovenia (20). The initial condition of the recursive formula (Eq. 3) varied according to the approach used for modeling the incidence rate (models 1-3 or raw data approach). The lower and upper limits of the 95% confidence interval of estimated prevalence rate were calculated on the basis of the Eq. 3 and separately using lower and upper limits of the 95% confidence interval (95% CI) of annual incidence rate at the plateau (from the period between 2004 and 2012).

Additionally, we predicted the prevalence rate for the year 2020. For this purpose the percentage of patients who reached the age of 19 after 2013 was calculated as the mean value of the previous three years (ie, 2011-2013). The incidence rate was assumed to be constant for the years 2013 to 2020, corresponding to the value of the incidence rate plateau reached in the period from 2004 to 2012. Additionally, we estimated the number of people aged 19 or younger for the year 2020 on the basis of a geometric growth model, where the geometric growth rate was constant and equaled to the mean growth rate in the previous three years (2011-2013). Tables, and graphs were made in Microsoft Excel® 2010 (Microsoft Corporation, Redmond, WA, USA).

## Results

### Modeling the annual incidence rate of ADHD in Slovenia

**Figure 1:**
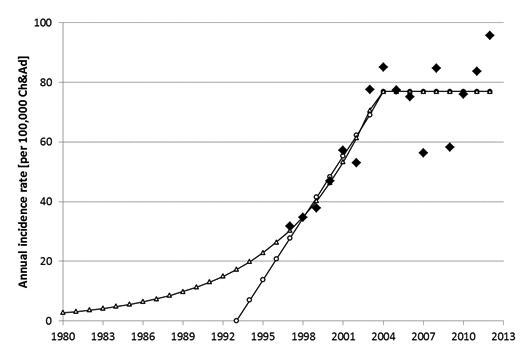
The annual Slovenian incidence rate of attention deficit hyperactivity disorder (ADHD) per 100 000 children and adolescents (Ch&Ad). Raw data (diamonds), linear model (line with circles), exponential model 2 (line with triangles).

The annual incidence rates of ADHD diagnosis in Slovenia increased from 1997 to 2003 (2.4-fold) (Table 1). Mean value of ADHD annual incidence rate between 2004 and 2012 was 77 (95% CI 67-87, n = 9). Epidemiological models were used for calculation of incidence rates (Figure 1). The calculated parameters of linear models were as follows: a = 27.67, b = 6.909, r^2^ = 0.875; exponential model: A = 30.36, k = 0.141, and r^2^ = 0.931.

**Table 1:** Annual incidence of attention deficit hyperactivity disorder (ADHD) obtained from national database and the Slovenian population data in the period from 1997 to 2013 (18,19)

Year	Crude incidence among children and adolescents	Number of children and adolescents	Number of 19-y olds	Proportion of 19-y olds (%)*	Incidence rate per 100 000 children and adolescents
**1997**	156	491,915	29,946	6.09	31.7
**1998**	166	479,240	30,505	6.37	34.6
**1999**	177	467,980	30,061	6.42	37.8
**2000**	214	456,145	29,730	6.52	46.9
**2001**	254	444,360	28,820	6.49	57.2
**2002**	230	433,884	27,472	6.33	53.0
**2003**	329	424,472	26,949	6.35	77.5
**2004**	354	415,850	26,661	6.41	85.1
**2005**	316	408,383	25,069	6.14	77.4
**2006**	303	403,028	25,880	6.42	75.2
**2007**	224	397,958	26,388	6.63	56.3
**2008**	334	394,304	24,955	6.33	84.7
**2009**	229	392,987	23,184	5.90	58.3
**2010**	299	393,176	22,898	5.82	76.0
**2011**	329	393,202	21,506	5.47	83.7
**2012**	378	394,681	20,669	5.24	95.8
**2013**		396,657	20,366	5.13	

*Proportion of 19-y-olds relative to the number of children and adolescents.

### The estimation of prevalence rate of ADHD in Slovenia

**Figure 2:**
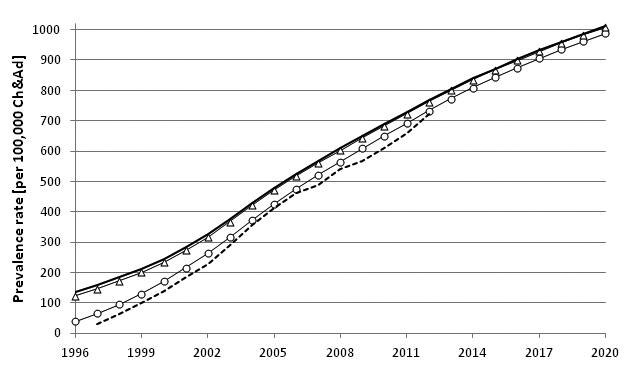
The point estimates of the prevalence rate of attention deficit hyperactivity disorder (ADHD) in Slovenia per 100 000 children and adolescents (Ch&Ad). Calculation based on raw incidence data (dashed line), linear model for incidence rate (line with circles), exponential incidence model from 1987 onwards (line with triangles), and exponential incidence model from 1980 onwards (solid line)

The point estimates of the prevalence rate calculated on the basis of 4 different models were used for calculation of the annual incidence rate (Figure 2). The deviation of the point estimates of the prevalence rate obtained by different models was relatively small: 6.0% for 2012. The mean prevalence of children and adolescents with ADHD in 2012 was 2950 (95% CI 2600-3300) or 750 (95% CI 660-840) per 100 000 children and adolescents. The predicted prevalence rate for 2020 was 1000 (95% CI 875-1125) per 100 000 children and adolescents or 1%, meaning that in 2020, 4050 (95% CI 3550-4550) children and adolescents will be diagnosed with ADHD.

The prevalence rate of ADHD diagnosis in Slovenia increased 4.8-fold from 1997 to 2012. In this period, the prevalence rate increased almost linearly.

The prevalence of ADHD diagnosis in 2020 was predicted to be 6.3-fold higher than in 1997, 1.3-fold higher than in 2012, and 1.8-fold higher than in 2007. All values were calculated using the exponential model with the starting point at 1980, because the differences within this model were smallest, although the differences among all the models were very small.

## Discussion

In the observed period, the annual incidence rates of ADHD diagnosis in Slovenia increased logarithmically and the prevalence rates increased linearly. The annual incidence increased 1.5-fold from 1997 to 2000 and 2.5-fold from 1997 to 2003. The prevalence of ADHD diagnosis increased 3.5-fold from 2000 to 2012 and 2.5-fold from 2000 to 2006. The increase in the incidence and prevalence in Slovenia is similar to that in Western countries (21) and could mainly be attributed to the implementation of new diagnostic criteria (ICD-10 and DSM-IV), which are more detailed than those used previously (ICD-9, DSM-III-R) (5,8,9). Second, the teachers and parents may be more sensitive to ADHD symptoms, which increases the number of referrals to specialist services (22). Third, in this period new drugs for ADHD became available (ie, nonstimulant atomoxetine and stimulant osmotic release oral delivery system methylphenidate), with pharmaceutical marketing in this field being increasingly active, which improved identification and reduced negative attitudes toward pharmacological treatment (23). ADHD was underdiagnosed in Slovenia in comparison with other EU countries. In Germany, the 2008 prevalence rate according to the ICD-10 criteria was 1.0% (the estimate is based on a representative sample of parents of 2452 children and adolescents aged 7-17 years), which is almost 2-fold higher than in Slovenia (11). In Spain, the 2011 prevalence rates of ADHD according to the ICD-10 in school-age children (6-12 years) were within the range of 1.2%, which is 2 times higher than in Slovenia (24). Similarly, in Great Britain the 2008 total prevalence of ADHD in children and adolescents aged from 13 to 17 years was 7.4%, which is about 10 times higher than in Slovenia (25). A systematic review found no significant differences in the prevalence of ADHD between Europe and North America (10).

Underdiagnosis in Slovenia might be attributable to the fact that only few psychiatrists diagnose ADHD and prescribe ADHD medicines. Also, there are no national guidelines for ADHD treatment and diagnosis, which would integrate the existing knowledge about ADHD into the Slovenian health care system (18,26). Parents and teachers also play a vital role and should be more extensively included in the ADHD recognition scheme in Slovenia (27).

In many countries, the main barrier to providing treatment of psychiatric disorders is the limited treatment in primary care settings (28). In Slovenia, this is particularly the case because of the high workload of Slovenian family physicians, meaning they are less likely to become involved in the treatment and prevention of mental health disorders, including ADHD (29,30). This leads to lower recognition and treatment of mental disorders and underprescription of psychiatric medicines (31). Slovenian psychiatry is predominantly hospital-based and thus poorly accessible. A program for the development of general community psychiatric services in Slovenia is in its beginnings, but will have to face all kinds of obstacles before it can be thoroughly implemented and improve access, quality, and comprehensiveness of psychiatric care according to modern standards of psychiatric service delivery (30). Another reason for ADHD underdiagnosis in Slovenia is a low percentage of urban population in Slovenia, as higher world prevalence rates of ADHD were found in urban areas (32).

The incidence rates calculated in our four models show the models’ usefulness in the calculation of incidence data from the NIPH database as well as their wide applicability. The incidence rate of ADHD diagnosis obtained from the model 1 was deviated, but primarily because of inaccuracies in the database. In prevalence calculation, there were minimal differences among the models, which showed the usefulness and appropriateness of these models to estimate the future ADHD prevalence rate. These models can be used in planning resources to cope with the burden of ADHD (16).

Although data from western countries show a fast increase in the number of ADHD patients in the last decade, in Slovenia this trend is observed with a big delay. However, this increase could be a result of improved diagnostics and awareness, rather than an increase in real prevalence. Therefore, this issue needs to be investigated in a new study including regional data.

There are several advantages of using claims data for an epidemiological study of ADHD, for example using longitudinal data from a large national population-based sample to calculate the annual incidence (32). However, this study has several limitations. First, we did not differentiate ADHD patients by disease subtypes. We defined ADHD as a chronic disorder in children and adolescents, which was not true in some cases, and it was also possible that some psychiatrists did not enter the correct diagnosis. Sex, and variables such as cultural background and socioeconomic status were not available in the database. The database included only initial data upon diagnosis, with no follow-up information. Moreover, the oscillation of the incidence data in the period from 2004 to 2012 was quite substantial, so we used the mean value of data from this period. On the other hand, the data from 1997 to 2003 were more consistent allowing us to develop two regression models: linear and exponential. Finally, there was no available registry of ADHD patients in Slovenia, limiting the possibilities of cross-national data comparison. In order to determinate the “real” prevalence rate of ADHD in children and adolescents we should have a register of patients with ADHD, which has not yet been established in Slovenia. We also did not calculate regional differences due to the small size of the country, small number of patients with ADHD, and variability of access to ADHD specialists, which were available mainly in the two largest cities.

In this study the annual incidence and prevalence rate of ADHD diagnosis were evaluated using four different approaches. All four approaches gave were very similar the results. The incidence and prevalence rate of children and adolescents with ADHD in Slovenia were increasing, but it was still 2 to 10 times slower than in some comparable countries. According to the calculated prevalence of ADHD in Slovenia, the diagnosis rates in children and adolescents did not exceeded those expected according to the rates in Western countries (less than 1% in children and adolescents). All models showed similar results, which indicated that they were appropriate for calculating ADHD incidence and prevalence using national database data. The epidemiological models could be used to assist ADHD epidemiology calculation in other countries in the region. Finally, these data indicated an urgent need for further research of health care utilization and quality of care in the field of ADHD in Slovenia.
